# Distinguishing space groups by electron channelling: centrosymmetric full-Heusler or non-centrosymmetric half-Heusler?

**DOI:** 10.1107/S2053273319016942

**Published:** 2020-02-19

**Authors:** Vidar Hansen, Andrey Kosinskiy, Johan Taftø

**Affiliations:** a Department of Mechanical and Structural Engineering and Materials Science, University of Stavanger, PO Box 8600, Stavanger 4036, Norway; b Department of Physics, Centre for Materials Science and Technology, University of Oslo, PO Box 1048, Blindern, Oslo N-0316, Norway

**Keywords:** space groups, inversion symmetry, electron channelling, X-ray emission, Heusler crystals

## Abstract

X-ray emission under electron-channelling conditions is used to distinguish between a non-centrosymmetric half-Heusler and a centrosymmetric full-Heusler crystal. For TiCo_1.5+*x*_Sn the space-group determination based on a Rietveld refinement procedure became challenging for increasing Co content (*x* > 0.2), while electron channelling proved successful for higher Co content (*x* = 0.35). This technique can be used on crystals as small as (10 nm)^3^.

## Abstract 2

Electron channelling was successfully used to determine the space group of a crystal where conventional diffraction failed to distinguish between half-Heusler and full-Heusler.

## 1. Introduction   

Centrosymmetric and non-centrosymmetric crystals may have the same extinctions of reflections. Potential experimental techniques to obtain structure-factor phase information, and thus to test for lack of centrosymmetry, are many-beam diffraction and standing-wave or channelling experiments with incident X-rays, neutrons or electrons. A more established technique to distinguish between centrosymmetric and non-centrosymmetric crystals is anomalous scattering of X-rays; see the pioneering work of Bijvoet *et al.* (1951 ▸) and, for example, Marezio (1965 ▸).

In this short communication, electron channelling is used experimentally to distinguish between a half-Heusler crystal with space group 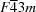 (No. 216) and a full-Heusler, 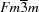 (No. 225). The Ti–Co–Sn system was studied. The specimen fabrication, starting with arc melting, is described by Kosinskiy *et al.* (2016 ▸). The composition of the Heusler phase was carefully measured using an energy-dispersive X-ray spectrometer attached to a scanning electron microscope. The composition was measured to be Ti_0.27_Co_0.49_Sn_0.24_ with an estimated average atomic content of Ti_4.00_Co_7.40_Sn_3.60_ within the unit cell, based on combining lattice-parameter measurements (Kosinskiy *et al.*, 2016 ▸) with application of Vegard’s law. This unit-cell content corresponds to the chemical formula Ti_1.00_Co_1.85_Sn_0.90_ in the usual way of expressing the atomic content of a Heusler compound.

In this study, electron channelling is used to obtain direct information about crystal symmetry and space groups. This is a task quite different from using electron channelling to locate atoms in crystals, the latter being referred to as the ALCHEMI technique (Spence & Taftø, 1983 ▸). Rather, it is an extension of using electron channelling to determine the polar direction of non-centrosymmetric crystals (Taftø, 1983 ▸; Jiang *et al.*, 2002 ▸).

## 2. Experimental details   

**Figure 1:**
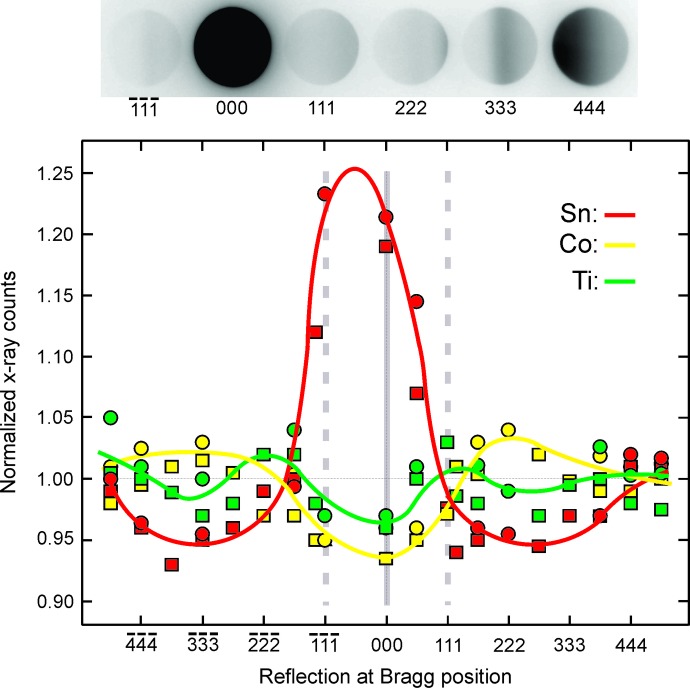
Normalized X-ray emission, as a function of the incident-beam direction, from the three elements of the crystal, namely the *L* line of Sn and the *K* lines of Ti and Co. The squares (red Sn, yellow Co and green Ti) and circles (red Sn, yellow Co and green Ti) are from two sets of measurements from different specimen regions. At the top is shown an example of the diffraction condition during the acquisition of an X-ray spectrum.

The electron-channelling experiments were performed with 200 keV incident electrons by operating a JEOL 2100 transmission electron microscope in the convergent-beam diffraction mode. The electron-induced element-characteristic X-ray emission signals were detected using an EDAX energy-dispersive spectrometer. Measurements were done under planar channelling conditions, with reflections along the (*hhh*) reciprocal-lattice row excited (Fig. 1 ▸). More details on electron channelling from Heusler crystals are presented in a recent paper by Hansen *et al.* (2018 ▸) and in another paper by Morimura & Hasaka (2006 ▸). A focused electron beam of diameter around 10 nm illuminated areas of thickness around 20 nm near the edge of a wedge-shaped region on the specimen. The convergence of the incident beam (divergence of the Bragg-reflected beams, Fig. 1 ▸) corresponds to a diameter close to the length of the *g* vector for the 111 reflection, (1^2^ + 1^2^ + 1^2^)^1/2^/6.06 Å = 0.29 Å^−1^, where 6.06 Å is the lattice parameter of the crystal. Small clean and smooth areas of specimen were prepared by crushing a sintered specimen.

## 3. Discussion and conclusions   

The normalized X-ray emission from the three elements in the crystal is also shown in Fig. 1 ▸ for different incident-beam directions. These are shown for two sets of measurements. We note a significant deviation from inversion across incidence parallel to the 111 planes, in particular by comparing the X-ray yields for the 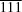 and 111 reflections at the Bragg position. Additional pairs of spectra with the 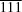 and 111 reflections at the Bragg position were acquired and the ratios are shown in Table 1 ▸. In all cases we note that the ratio for Sn is below unity, the ratio for Ti is above unity and the ratio for Co is close to unity.

**Figure 2:**
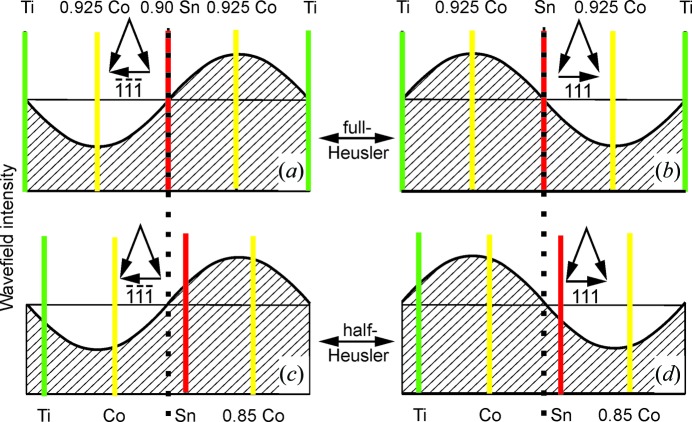
(*a*), (*b*) Two-beam calculations of the wavefield modulation for the centrosymmetric model, space group 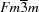 (No. 225), with the Bragg condition fulfilled for (*a*) the 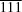 reflection and (*b*) the 111 reflection. (*c*), (*d*) Similarly for the non-centrosymmetric model, space group 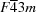 (No. 216). In the analytical calculations for both the centrosymmetric and non-centrosymmetric models, the origin is chosen at the same position within the figure (vertical dotted line). The origin for both models is at the position resulting in a real and positive structure factor for the 111 reflection (and thus also for the 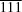 reflection). This origin is in the Sn plane in the centrosymmetric model and the crystal had to be shifted by 25°/360° parts on the repetition unit for the non-centrosymmetric model.

Assuming the two-beam approximation applies, the electron wavefield intensity across the repetition unit behaves as indicated in Fig. 2 ▸ for the 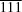 and 111 reflections at the Bragg position. The amplitudes of these curves vary with thickness, as seen from the analytical expression for wavefield modulation in the two-beam approximation with absorption (diffuse scattering) (Taftø, 1983 ▸),
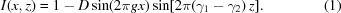
Here, *z* is the distance from the entrance surface, *x* the position within the repetition unit and (γ_1_ − γ_2_) the inverse of the period of the thickness oscillation. *D* is a damping factor due to diffuse scattering out of the two beams (direct and Bragg-reflected beams), the averaging over the thickness of the crystal area which is illuminated by the incident electrons, and the convergence of the incident beam.

In the two-beam approximation the wavefield shows the same modulation, whether half-Heusler or full-Heusler. Relative to this modulation the crystal is shifted (Fig. 2 ▸). The origin, which we choose to place in the Sn plane for the full-Heusler model, is for the half-Heusler model at the position maintaining a real and positive value of the structure factor of the 111 reflection, and thus also the 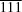 reflection.

Although not relevant for the space-group issue, we add the following comments. From the modulation calculated in Fig. 2 ▸ it is expected that the ratio 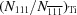 should be the inverse of 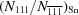. The average ratio for Sn is 0.87 (Table 1 ▸) and the inverse is 1.15. The observed ratio for Ti is only 1.07, however, which may, at least partly, be explained by the presence of around 10% of the Ti atoms on the Sn position.

In a recent study of the Heusler system TiCo_1.5+*x*_Sn (Kosinskiy *et al.*, 2016 ▸), it was reported that it became exceedingly difficult from Rietveld refinement of X-ray powder diffraction data to distinguish between the non-centrosymmetric half-Heusler and the centrosymmetric full-Heusler for *x* > 0.2. In the present study a clear lack of symmetry is observed for a crystal approaching the composition of a full-Heusler (*x* = 0.35). We conclude that the crystal is a non-centrosymmetric half-Heusler with space group 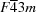 (No. 216). This demonstrates that electron channelling, a technique that can be used on crystal volumes as small as (10 nm)^3^, is highly sensitive to lack of centrosymmetry.

## Figures and Tables

**Table 1:** The ratio between the X-ray emission, 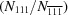, from seven pairs of additional measurements with the 111 and 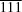 reflections at the Bragg position, respectively

Sn	0.81	0.91	0.91	0.84	0.89	0.86	0.83
Ti	1.09	1.07	1.08	1.08	1.03	1.08	1.07
Co	1.03	1.00	0.99	1.03	1.02	1.02	1.03
